# Sunitinib treatment enabling resection of massive liver metastasis: a case report

**DOI:** 10.1186/1752-1947-7-234

**Published:** 2013-10-03

**Authors:** Shingo Mitomo, Takeshi Takahara, Hiroyuki Nitta, Tomohiro Fujita, Naoko Ito, Noriyuki Uesugi, Tamotsu Sugai, Go Wakabayashi

**Affiliations:** 1 Department of Surgery, Iwate Medical University School of Medicine, Morioka, Iwate 020-8505, Japan; 2 Division of Molecular Diagnostic Pathology, Department of Pathology, Iwate Medical University School of Medicine, Morioka, Iwate 020-8505, Japan

**Keywords:** Chemotherapy, Liver damage, Liver metastasectomy, Liver metastasis, Renal cell carcinoma, Sunitinib

## Abstract

**Introduction:**

Sunitinib was developed as a molecular-targeted drug to treat advanced renal cell carcinoma. It is not yet known whether liver damage occurs in patients with liver metastases of renal cell carcinoma after sunitinib administration. Here, we report the case of a patient with an inoperable massive liver metastasis of renal cell carcinoma for whom sunitinib administration was dramatically effective with no obvious evidence of liver damage. As a result, the liver metastasis could be resected. We emphasize the dramatic reduction in liver metastasis with sunitinib treatment, and the histopathological effects of sunitinib on the non-tumorous liver parenchyma.

**Case presentation:**

A 54-year-old Japanese woman was diagnosed with right renal cell carcinoma and underwent right nephrectomy 12 years earlier. She presented to a local clinic with right abdominal pain. A computed tomography scan showed a massive liver metastasis occupying her right hepatic lobe, and she was referred to our hospital for treatment. The diagnosis was not only liver metastasis, but also left renal metastasis. Oral administration of tyrosine kinase inhibitor sunitinib was started. Adverse events due to sunitinib included liver dysfunction, thrombocytopenia, and decreased hemoglobin, but she completed eight courses with the help of drug holidays and dose adjustments. Post-treatment computed tomography showed a dramatic reduction in size of her liver metastasis, enabling right lobectomy of her liver. Histopathological findings showed no obvious liver damage due to chemotherapy in non-cancerous parenchymal areas.

**Conclusions:**

With the availability of sunitinib, some patients with potentially unresectable massive liver metastases of renal cell carcinoma may be able to undergo major hepatectomy curatively and safely with little histopathological damage to non-tumorous liver parenchyma, thus improving their prognosis.

## Introduction

Following the introduction of new anticancer agents in recent years, multimodal therapy incorporating surgical resection for metastatic liver cancer has been reported to be effective [1]. However, some cases in which chemotherapy caused liver damage have also been reported. Oxaliplatin, which is frequently used to treat liver metastases of colon cancer, characteristically causes sinusoidal dilatation, whereas irinotecan causes nonalcoholic steatohepatitis (NASH) [2]. Sunitinib and sorafenib, which were developed as molecular-targeted drugs to treat advanced renal cell carcinoma, mainly block vascular endothelial growth factor and platelet-derived growth factor receptor tyrosine kinases, and thereby inhibit tumor growth and angiogenesis. Sunitinib was approved by the United States Food and Drug Administration in 2006 and was launched in Japan in June 2008 as a drug for the treatment of inoperable or metastatic renal cell carcinoma and imatinib-resistant gastrointestinal stromal tumor (GIST). A metastasectomy in renal cell carcinoma after neoadjuvant therapy with sunitinib [3], and the use of sunitinib for a patient with GIST in the neoadjuvant setting to achieve complete surgical resection [4] has been reported. Whether liver damage occurs in patients with liver metastases of renal cell carcinoma after sunitinib administration is not yet known. Here, we report a case in which sunitinib administration was dramatically effective with no obvious evidence of liver damage in a patient with an inoperable massive liver metastasis of renal cell carcinoma. Treatment by sunitinib enabled subsequent hepatectomy to be performed safely.

We emphasize the dramatic reduction in liver metastasis with sunitinib treatment, and the histopathological effects of sunitinib on the non-tumorous liver parenchyma.

## Case presentation

**Figure 1:**
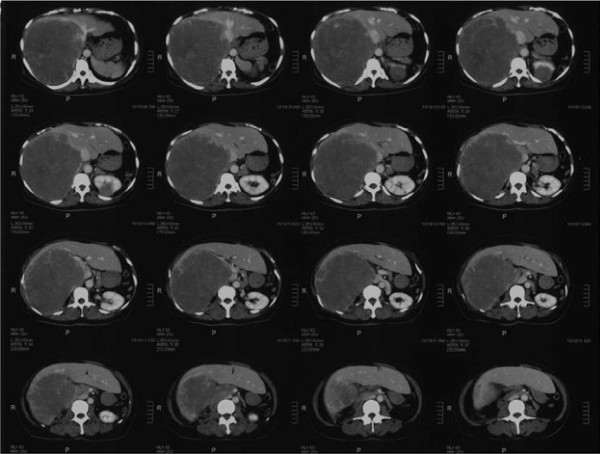
**Abdominal computed tomography before sunitinib administration.** A massive liver metastasis is present in the right hepatic lobe, and there is also a left renal metastasis.

**Figure 2:**
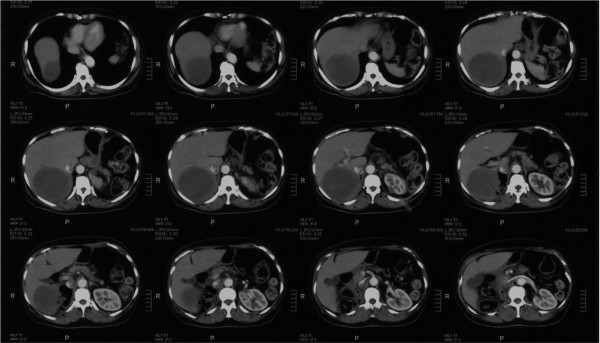
**Abdominal computed tomography after sunitinib administration.** The liver metastasis has liquefied and shrunk dramatically.

**Figure 3:**
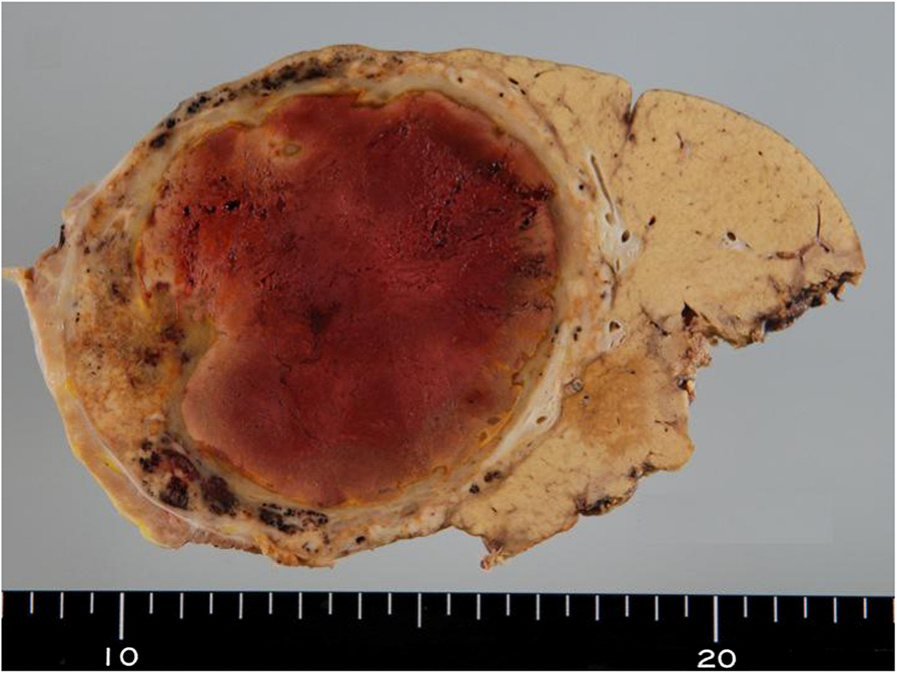
**Excised specimen.** The tumor is covered in a capsule, with widespread necrosis inside.

A 54-year-old Japanese woman was diagnosed with right renal cell carcinoma, and she underwent right nephrectomy in the Department of Urologic Surgery at our hospital. The size of her tumor was 80mm. A diagnosis of T2N0M0 Stage II was made based on histopathological findings. The histological subtype of the renal cell carcinoma was clear cell. After 12 years she began to feel right abdominal pain and was examined at a local clinic. A computed tomography (CT) scan showed a massive liver metastasis occupying her right hepatic lobe, as well as a left renal metastasis. She was referred to the Department of Urologic Surgery at our hospital for treatment, and oral sunitinib was started. A pre-treatment abdominal CT showed a massive liver metastasis measuring 22cm × 17cm in her right hepatic lobe, and a left renal metastasis of which the largest diameter was 4cm. Neither ascites nor lymph node metastases were present (Figure 1). The tumor markers carcinoembryonic antigen and carbohydrate antigen 19-9 were both within normal limits. Sunitinib was administered with the standard regimen of 50 mg/day for 4 weeks followed by a 2-week drug holiday. According to the National Cancer Institute Common Terminology Criteria for Adverse Events version 4.0, adverse events during oral administration included grade 3 liver dysfunction, thrombocytopenia, and decreased hemoglobin during course 1; grade 2 neutropenia during course 6; and grade 2 renal dysfunction and hypothyroidism at the end of course 8. However, with the introduction of drug holidays, dose adjustments (37.5mg to 50mg/day, increased or decreased according to the severity of side effects), and changes in the administration method (from four doses/two holidays to two doses/two holidays), the patient was able to complete the eight courses. A CT scan after the completion of the eight courses of sunitinib showed that her liver metastasis had shrunk dramatically to a long diameter of 9cm. Her left renal metastasis had shrunk to a long diameter of 1cm (Figure 2). According to the guidelines of the Response Evaluation Criteria in Solid Tumours [5], a 73% decrease in the size of a tumor of the liver is defined as partial remission. The preoperative indocyanine green retention rate at 15 minutes was 18%. Based on these results, right lobectomy of her liver was performed to remove the renal cell carcinoma liver metastasis, which had been dramatically reduced by sunitinib. During surgery, laparotomy was performed using a J-shaped incision. Her right hepatic artery and the right branch of her portal vein were transected in that order, and hepatic parenchyma resection was performed using a combination of the liver hanging maneuver and an anterior approach. Next, her right hepatic duct was transected. Tumor thrombosis was present in her right hepatic vein, and this was excised as far as possible. Her liver was normal, showing no visible effects of chemotherapy. The excised specimen measured 92mm × 78mm. The tumor was covered by a capsule, and its interior showed widespread brownish coloration, indicating necrosis (Figure 3).

**Figure 4:**
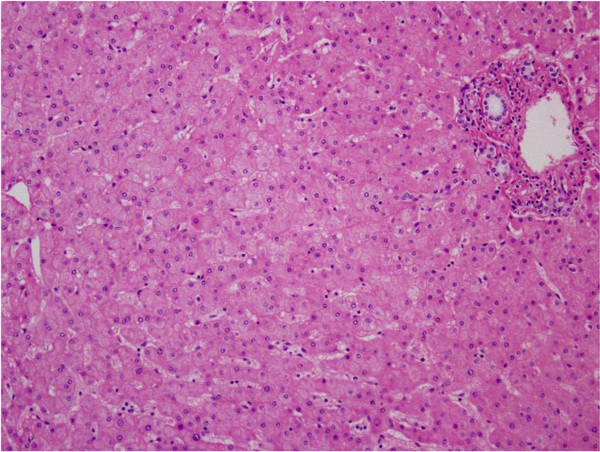
**Histopathological findings of background liver (hematoxylin and eosin staining ×100).** No sinusoidal dilatation or steatohepatitis is present.

Immunohistopathology showed that her tumor was CD10(+), cytokeratin (CK)7(+), and CK20(-), and a diagnosis of renal cell carcinoma liver metastasis was made. Hematoxylin and eosin staining of her non-tumorous liver tissue showed no sinusoidal dilatation, with a Rubbia-Brandt score of 0, and the nonalcoholic fatty liver disease (NAFLD) activity score was 1, with no obvious steatohepatitis (Figure 4).

The patient’s postoperative course was uneventful, and she was discharged after 13 days. Radiofrequency ablation was performed on her left renal metastasis at 1 month after discharge. At the time of writing, 1 year after surgery, no recurrence has been observed in either her liver or her left kidney.

## Discussion

Multiple liver metastases are frequently seen when renal cell carcinoma metastasizes to the liver; the frequency of solitary metastasis is 2% to 4% [6]. For this reason, there have been few reports of resection of liver metastatic foci. There is no single consistent view on the surgical indications for renal cell carcinoma liver metastasis. Stief *et al.*[7] performed hepatectomy for 13 patients with liver metastases, four of whom died from complications, and they reported that patients should be selected carefully. Staehler *et al.*[8] performed a retrospective comparative analysis of 88 patients with renal cell carcinoma liver metastases; liver metastases were resected in 66 and not resected in 20, forming the control group. The 5-year survival was 62.2% in the resection group and 29.3% in the control group. The authors stated that liver metastasis resection can be regarded as a valuable tool in the treatment of metastatic liver carcinoma, and metastatic foci should be resected when technically possible.

The frequency of adverse events during sunitinib treatment tends to be higher in Japanese patients than in Western patients [9], and drug holidays are obligatory in many cases. Because longer drug holidays are unwelcome from the viewpoint of limiting disease progression, it is very important to devise ways of adjusting dosage and methods of use to ensure that drug holidays do not exceed the minimum necessary. Reported side effects include thrombocytopenia, leukopenia, hypothyroidism, cardiac dysfunction, hand-and-foot syndrome, skin damage, diarrhea, and hypertension. Since side effects frequently appear early on, careful monitoring is required. In the present case, grade 3 liver dysfunction, thrombocytopenia, and decreased hemoglobin were present during course 1; grade 2 neutropenia occurred during course 6; and grade 2 renal dysfunction and hypothyroidism were seen at the end of course 8. However, with the inclusion of drug holidays, dose adjustments, and changes in the administration method, the patient was able to complete eight courses. We considered the renal toxicity of sunitinib treatment might be caused by only one kidney. In this case both renal and liver dysfunctions were transient, and recovered through the introduction of drug holidays and dose adjustments.

Multimodal therapy incorporating new anticancer agents for metastatic liver cancer is highly effective for liver metastases of colon cancer in particular but, at the same time, the liver damage caused by regimens including oxaliplatin or irinotecan means that caution is required during surgical hepatectomy. To investigate whether liver damage occurred during the use of sunitinib in the present patient, histopathological evaluation of sinusoidal dilatation in non-tumorous liver tissue was carried out by scoring according to the method of Rubbia-Brandt *et al.*[10], and the NAFLD activity score was used to evaluate NASH by the method of Kleiner *et al.*[11]. The liver was almost normal, with a sinusoidal dilatation score of 0 and an NAFLD activity score of 1, indicating no liver damage due to sunitinib.

There are very few reports of patients with massive liver metastases in whom liver metastasis resection has been performed after sunitinib administration. Although this means that it is still unclear whether sunitinib causes liver damage, in the present case, sunitinib was dramatically effective, enabling hepatectomy to be performed with no complications. Hepatectomy can also be regarded as a valuable tool from an oncological standpoint. The present histopathological results for the effects of sunitinib on the non-tumorous liver tissue might not be the same in all cases; further investigations involving a greater number of cases are required.

## Conclusions

In conclusion, with the availability of sunitinib, some patients with potentially unresectable massive liver metastases of renal cell carcinoma may be able to undergo major hepatectomy curatively and safely with little histopathological damage to non-tumorous liver parenchyma, thus improving their prognosis.

## Consent

Written informed consent was obtained from the patient for publication of this case report and accompanying images. A copy of the written consent is available for review by the Editor-in-Chief of this journal.

## Competing interests

The authors declare that they have no competing interests.

## Authors’ contributions

HN and TT designed the manuscript; SM wrote the manuscript; GW, HN, and TT performed the operation; NI, TF, and SM cared for the patient; and TS and NU made the pathological diagnosis. GW, supervised and approved the final manuscript. All authors read and approved the final manuscript.
